# A META-ANALYSIS OF EXTRACELLULAR VESICLES BIOMARKERS IN THE DIAGNOSIS OF ALZHEIMER’S DISEASE AND RELATED DEMENTIAS

**DOI:** 10.1093/geroni/igae098.4336

**Published:** 2024-12-31

**Authors:** Hash Brown Taha

**Affiliations:** University of Southern California, Los Angeles, California, United States

## Abstract

Accurate differential diagnosis of dementia disorders including Alzheimer’s disease (AD), frontotemporal dementia (FTD), dementia with Lewy bodies (DLB), Parkinson’s disease dementia (PDD), and vascular cognitive impairment and dementia (VCID), along with conditions like prodromal mild cognitive impairment (MCI) or negative controls (NCs), continues to challenge neurologists. Extracellular vesicles (EVs) have emerged as a popular tool for their capacity to encapsulate disease-specific signatures, particularly in neurodegenerative and neurological disorders. To this end, we have performed a comprehensive, PRISMA-guided systematic review and meta-analysis, utilizing sophisticated statistical modeling to determine the diagnostic accuracy, explore between-study variance and heterogeneity (I2), and investigate potential publication bias using biomarkers derived from general EVs (n = 44) or speculative CNS-enriched EVs (n = 18). For all comparative analyses, biomarkers derived from general EVs demonstrated superior diagnostic accuracy, less between-study variance, heterogeneity, and publication bias than those from speculative CNS-enriched EVs. The diagnostic accuracy was low in differentiating among different dementia disorders or from NCs. However, the analysis for diagnosing persons with AD vs. VCID achieved the highest diagnostic accuracy, suggesting that further studies may focus on this comparison. Meta-regressions revealed that studies using cerebrospinal fluid, employing citrate instead of EDTA for blood collection, using thrombin for coagulation factor depletion, isolating EVs with purer methods such as combined UC and SEC, not freezing EVs post-isolation, and quantifying miRNA biomarkers instead of proteins, achieved better diagnostic accuracy and less heterogeneity.

